# Unexpected Hepatitis B Virus Infection After Liver Transplantation — United States, 2014–2019

**DOI:** 10.15585/mmwr.mm7027a1

**Published:** 2021-07-09

**Authors:** Danae Bixler, Pallavi Annambhotla, Martha P. Montgomery, Tonya Mixon-Hayden, Ben Kupronis, Marian G. Michaels, Ricardo M. La Hoz, Sridhar V. Basavaraju, Saleem Kamili, Anne Moorman

**Affiliations:** ^1^Division of Viral Hepatitis, National Center for HIV/AIDS, Viral Hepatitis, STD, and TB Prevention, CDC; ^2^Office of Blood, Other Organ, and Tissue Safety, National Center For Emerging and Zoonotic Infectious Diseases, CDC; ^3^University of Pittsburgh Medical Center, Pennsylvania; ^4^University of Texas Southwestern Medical Center, Dallas.

Unexpected donor-derived hepatitis B virus (HBV) infection is defined as a new HBV infection in a recipient of a transplanted organ from a donor who tested negative for total antihepatitis B core antibody (total anti-HBc), hepatitis B surface antigen (HBsAg), and HBV DNA* before organ procurement. Such infections are rare and are associated with injection drug use among deceased donors (*1*). During 2014–2019, CDC received 20 reports of HBV infection among recipients of livers from donors who had no evidence of past or current HBV infection. Investigation included review of laboratory data and medical records. Fourteen of these new HBV infections were detected during 2019 alone; infections were detected a median of 38 (range = 5–116) weeks after transplantation. Of the 14 donors, 13 were hepatitis C virus (HCV)–seropositive^†^ and had a history of injection drug use within the year preceding death, a positive toxicology result, or both. Because injection drug use is the most commonly reported risk factor for hepatitis C,^§^ providers caring for recipients of organs from donors who are HCV-seropositive or recently injected drugs should maintain awareness of infectious complications of injection drug use and monitor recipients accordingly (*2*). In addition to testing for HBV DNA at 4–6 weeks after transplantation, clinicians caring for liver transplant recipients should consider testing for HBV DNA 1 year after transplantation or at any time if signs and symptoms of viral hepatitis develop, even if previous tests were negative (*2*).

All suspected unexpected cases of donor‐derived hepatitis B in the United States are reported to the Organ Procurement and Transplantation Network for review by the Ad Hoc Disease Transmission Advisory Committee. Suspected cases are referred to CDC to investigate whether donor-derived disease transmission occurred and identify interventions to prevent transmission and improve outcomes (*1*,*2*). Confirmed cases were defined as unexpected, new,^¶^ reproducible laboratory evidence of HBV infection (HBsAg or HBV DNA) occurring in liver recipients after transplantation that were reported to CDC during 2014–2019. All recipients who received organs from the same donor as the liver recipient were evaluated for donor-derived HBV infection using the same criteria. Available archived donor serum, plasma, or liver biopsy samples were tested for HBV DNA. State and local health departments shared information about recipient behavioral risk factors and outbreaks of health-care–associated HBV infection.

During 2014–2019, CDC investigated 30 suspected cases of unexpected, donor-derived HBV infection among liver recipients. Ten suspected cases were excluded because the recipients had nonreproducible HBV DNA (six), or false-positive total anti-HBc (two) or HBsAg (two) results. Twenty confirmed cases were included.

Median age at death of the 20 donors was 31 years (range = 20–46 years); 11 were male, and 19 were White. The most common cause of death was drug intoxication. Injection drug use and positive toxicology were each reported for 18 donors (Table). Sixteen donors, including 13 of 14 reported in 2019, were HCV antibody (anti-HCV)–seropositive; among these 13 donors, 12 had positive drug toxicology, 12 had a history of injection drug use, and 11 had both. Stimulants (cocaine or amphetamines) were the most common substances identified by toxicology screening. HBV DNA was detected in one archived donor serum sample and one archived liver biopsy specimen.

**TABLE:** Demographic and clinical characteristics and risk behaviors of deceased organ donors* reported to CDC because of hepatitis B virus infection in liver transplant recipients after transplantation — United States, 2014–2019

Characteristic	Yr of report to CDC, no. (%)	
	2014–2018 (N = 6)	2019 (N = 14)
**Age**		
Mean age, yrs (median)	27 (23)	33 (32)
Age range, yrs	20–43	20–46
Age, interquartile range, yrs	21–29	27–41
**Year, no. of deaths**		
2013	1	0
2014	0	0
2015	1	0
2016	3	0
2017	0	2
2018	1	10
2019	0	2
**Sex**		
Male	4 (67)	7 (50)
Female	2 (33)	7 (50)
**Race**		
White	6 (100)	13 (93)
Black or African American	0 (—)	1 (7)
**Risk factor for hepatitis B^†^ within the 12 mos before organ donation**		
Injection drug use	6 (100)	12 (86)
Incarceration (lockup, jail, prison, or a juvenile correctional facility) for >72 hours	5 (83)	8 (57)
Sex with a person who injected drugs by intravenous, intramuscular, or subcutaneous route for nonmedical reasons	4 (67)	3 (21)
Sex with a person who had sex in exchange for money or drugs	3 (50)	0 (—)
Sex with a person who had a positive test for, or was suspected of having, hepatitis B, hepatitis C, or HIV	1 (17)	0 (—)
Sex in exchange for money or drugs	1 (17)	0 (—)
Diagnosis or treatment for syphilis, gonorrhea, chlamydia, or genital ulcers during the preceding 12 months	1 (17)	0 (—)
Men who have sex with men, no. (% of males)	0 (—)	1 (14)
No history from next-of-kin	0 (—)	1 (7)
Developmental disabilities and long-term group home residence	0 (—)	1 (7)
**Toxicology screening**		
Amphetamines	4 (67)	6 (43)
Opiates	5 (83)	7 (50)
Benzodiazepines	4 (67)	4 (29)
Cannabinoids or Delta-9 tetrahydrocannabinol	1 (17)	7 (50)
Cocaine	1 (17)	8 (57)
Barbiturates	1 (17)	1 (7)^§^
PCP (phencyclidine)	0 (—)	1 (7)
Positive screen for any substance	5 (83)	13 (93)^¶^
Positive screen for any stimulant (cocaine or amphetamines)	4 (67)	11 (79)
**Cause of death**		
Drug intoxication	3 (50)	11 (79)
Trauma	1 (17)	2 (14)
Asphyxiation	1 (17)	1 (7)
Cardiovascular disease	1 (17)	0 (—)
Antemortem test results**		
Anti-HCV–positive (serum) (i.e., seropositive)	3 (50)	13 (93)
HCV RNA–positive (serum) (i.e., viremic)	0 (—)	9 (64)
**Archived specimen testing^††^**		
Plasma/serum tested for HBV DNA	5 (83)	9 (64)
Plasma/serum positive for HBV DNA	0 (—)	1 (7)^§§^
Splenocytes tested for HBV DNA	1 (17)	4 (29)
Splenocytes positive for HBV DNA	0 (—)	0 (—)
Liver biopsy specimen tested for HBV DNA	1 (17)^¶¶^	1 (7)***
Liver biopsy specimen positive for HBV DNA	1 (17)	0 (—)

**Abbreviations:** anti-HCV = antibody (IgG) to hepatitis C virus; HBsAg = hepatitis B surface antigen; HBV = hepatitis B virus; HCV = hepatitis C virus; total anti-HBc = total antibody to hepatitis B core antigen.

* Donors were included in the study if they had been reported to CDC during 2014–2019 and had negative total anti-HBc, HBsAg, and HBV DNA, and a liver recipient experienced new, reproducible laboratory evidence of HBV infection after transplantation.

^†^ Includes risk behaviors and other risk factors as defined in https://www.cdc.gov/mmwr/volumes/67/rr/rr6701a1.htm and https://pubmed.ncbi.nlm.nih.gov/23814319/. Behavioral risk factors were identified through next-of kin interviews or review of medical records. No donor met any of the following United States Public Health Service criteria: a woman who had sex with a man with a history of having had sex with men during the preceding 12 months; a child who was aged <18 months and born to a mother known to be infected with, or at increased risk for, HBV, or HCV infection; a child who had been breastfed within the preceding 12 months and the mother was known to be infected with, or at increased risk for, HIV infection; persons who had been on hemodialysis during the preceding 12 months (for hepatitis C only); or hemodilution.

^§^ Barbiturates had been prescribed for one included donor.

^¶^ Includes one donor with only prescribed barbiturates and 12 donors with a median of three substances (range = one to four).

** Routine antemortem donor test results as recommended in https://pubmed.ncbi.nlm.nih.gov/23814319/. All HIV test results were negative.

^††^ All archived specimens were tested during investigation of suspected donor-derived transmission of HBV infection. Testing was performed at CDC’s Division of Viral Hepatitis Laboratory, unless otherwise specified.

^§§^ The positive result was obtained by the Public Health Ontario Laboratory, Ontario, Canada.

^¶¶^ Archived liver biopsy specimen taken from the recipient 1 week after transplantation.

*** Archived reperfusion liver biopsy specimen.

New HBV infection was identified in 18 liver and two liver-kidney recipients at a median of 41 weeks after transplantation (range = 5–116 weeks). Among cases reported during 2019, hepatitis B test conversion was first identified at a median of 38 weeks after transplantation (Figure). None of 31 recipients of nonliver organs** from the 20 donors developed a new infection with hepatitis B. No behavioral risk factors or health care–associated hepatitis B outbreaks were reported in association with any case. Hepatitis B vaccination status was unavailable for the majority of recipients.

**FIGURE:**
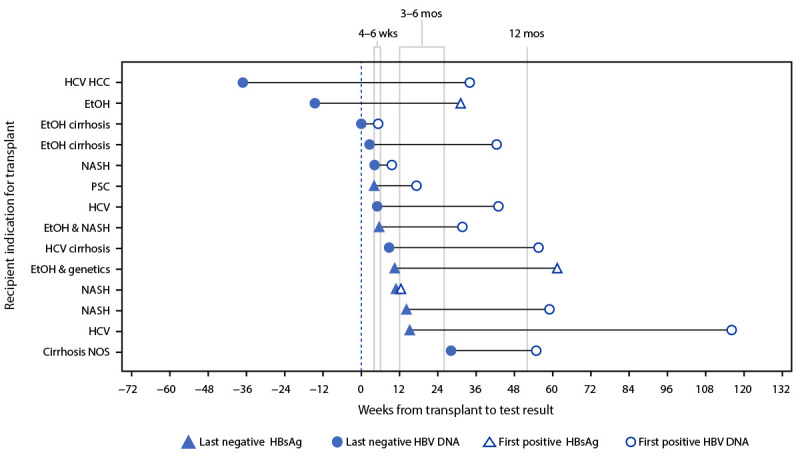
Timing of last negative and first positive test for hepatitis B virus among liver recipients with hepatitis B virus test conversion after transplantation reported to CDC — United States, 2019 **Abbreviations:** EtOH = alcohol(ic); HBsAg = hepatitis B surface antigen; HBV = hepatitis B virus; HCC = hepatocellular carcinoma; HCV = hepatitis C virus; NASH = nonalcoholic steatohepatitis; NOS = not otherwise specified; PSC = primary sclerosing cholangitis.

## Discussion

HBV infection among transplant recipients can occur from reactivation of previous HBV infection (*3*), primary infection after transplantation, or donor-derived transmission (*1*). This report provides evidence that transmission of HBV from donors occurred despite negative organ donor HBV DNA, HBsAg, and total anti-HBc results before organ procurement. Among 14 cases reported during 2019, all donors but one were HCV-seropositive with a history of injection drug use, a positive toxicology result, or both. Clinicians caring for liver recipients, particularly those from donors with positive anti-HCV serology or a history of injection drug use, should maintain awareness of delayed HBV presentation and consider testing for HBV DNA at 1 year after transplantation or at any time if signs and symptoms of viral hepatitis develop, even if prior tests were negative (*2*).

Donors might have been exposed to HBV through injection drug use shortly before death; thus, organ procurement might have occurred during the eclipse period,^††^ before HBV DNA was detectable in donor serum. During the eclipse period, HBV enters the hepatocyte nucleus and forms covalently closed circular DNA, which endures throughout the life of the nondividing hepatocyte (*4*). Therefore, liver recipients should be more likely than nonliver organ recipients to experience HBV infection from donors with eclipse period infection. An alternative hypothesis is that HCV coinfection suppressed HBV replication in certain donors, resulting in occult HBV infection. In 20% of HBV/HCV coinfections, patients can test negative for all HBV serum markers (*5*). Subsequent immunosuppression or treatment for HCV infection among liver recipients might lead to reactivation of HBV infection (*5*) after transplantation. The observed interval (median = 41 weeks) between transplantation and diagnosis of HBV infection in these cases is similar to the prolonged interval between transplantation and reactivation of hepatitis B infection among recipients of a liver from a donor who was total anti-HBc seropositive(*3*).

In the United States, liver transplants from HCV-seropositive donors increased from 308 in 2014 to 644 in 2018, and liver transplants from HCV RNA-positive donors increased from 236 in 2015 to 418 in 2018 (*6*). The national rate of drug overdose deaths per 100,000 population^§§^ increased during 2012–2018 from 1.4 to 4.5 for cocaine, and from 0.8 to 3.9 for psychostimulants, including amphetamines (*7*). Deaths related to synthetic opioids also increased during that time frame (*7*).^¶¶^ Injection of cocaine (*8*) or methamphetamine (*9*) and high-risk sexual behavior (*8*) have been reported in association with hepatitis B outbreaks. These data indicate that the increased number of unexpected donor-derived HBV infections among liver recipients during 2019 might be related to changes in patterns of stimulant use and associated behaviors, or to increased transplantation of organs from anti-HCV–seropositive donors who injected drugs. The most common risk factor for hepatitis B and hepatitis C is injection drug use.

The findings in this report are subject to at least four limitations. First, detection of infection after transplantation is dependent on testing and reporting by transplant centers. The 2013 Public Health Service guidelines (*10*) recommended risk-based recipient screening for hepatitis B after transplantation. However, the timing and frequency of recipient testing after transplantation might have varied during the timeframe of this study by year, transplant center, organ type, or the donor’s hepatitis C status. The impact on these findings cannot be quantified but might result in underestimation of donor-derived HBV infections. Second, previous recommendations (*10*) did not specify how hepatitis B testing of recipients should be accomplished before transplantation. Because of incomplete test results before transplantation, the presence of resolved or occult HBV infection before transplantation cannot be ruled out for certain recipients. Third, archived liver biopsy specimens were unavailable for the majority of donors. If stored correctly, liver tissue is the most likely specimen to have detectable HBV DNA during the eclipse period, which might confirm donor-derived infection. Finally, despite efforts to ascertain risk factors, risk behaviors for organ recipients might have been underreported, resulting in overestimation of donor-derived infections.

Early detection of donor-derived HBV infection is important for preventing hepatitis B–related complications among organ recipients and unintended transmission to their contacts. Recipients should be offered hepatitis B vaccination and hepatitis B testing (including total anti-HBc, HBsAg, and HBV surface antibody) before transplantation and HBV DNA testing at 4–6 weeks after transplantation (*2*). Additional testing for HBV DNA 1 year after transplantation (*2*) should be considered for liver transplant recipients, especially if the donor had risk factors for hepatitis B, including injection drug use or positive HCV serology. Recipients with signs or symptoms of liver injury after transplantation should be tested for viral hepatitis, even if previous hepatitis B or hepatitis C testing was negative (*2*). More broadly, providers caring for recipients of organs from donors who recently injected drugs or are HCV-seropositive should maintain awareness of infectious complications of drug use and monitor recipients accordingly.

**Summary**

**What is already known about this topic?**

Unexpected donor-derived hepatitis B virus (HBV) infection after organ transplantation is rare and is associated most commonly with donor injection drug use.

**What is added by this report?**

During 2019, the Organ Procurement and Transplantation Network and CDC received an increased number of reports of HBV infection among liver recipients from HBV-negative donors; 12 of 14 implicated donors had evidence of recent injection drug use, and 13 donors were hepatitis C virus (HCV)–seropositive.

**What are the implications for public health practice?**

Providers caring for recipients of organs from donors who are HCV–seropositive or who recently injected drugs should maintain awareness of infectious complications of drug use and monitor recipients accordingly.

## Footnotes

* HBsAg and HBV DNA are laboratory evidence of current infection with HBV. Total anti-HBc indicates past or current infection with HBV. Recipients of a liver from a donor with isolated total anti-HBc positive results can develop reactivation of hepatitis B after transplantation.

† Donors who are HCV-seropositive include both HCV-viremic (anti-HCV–seropositive and HCV RNA–positve) and HCV-nonviremic (anti-HCV–seropositive and HCV RNA–negative) donors as described in https://pubmed.ncbi.nlm.nih.gov/28556422/.

§
https://www.cdc.gov/hepatitis/statistics/SurveillanceRpts.htm

¶ New infection with HBV is defined as a positive viral detection test (HBsAg or HBV DNA) in an organ recipient without evidence for HBV infection (anti-HBc, HBsAg, or HBV DNA) preceding transplantation.

** Analysis included nonliver organ recipients without evidence of hepatitis B infection (total anti-HBc, HBsAg, or HBV DNA) before transplantation. Twenty single kidney, six heart, and four bilateral lung recpients and one kidney-pancreas recipient received negative test results for HBsAg or HBV DNA after transplantation at the time of the investigation. Seven nonliver organ recipients with previous evidence of hepatitis B infection were excluded from this analysis, including six single kidney recipients and one bilateral lung recipient.

†† Eclipse period is defined as the 1–12 weeks between exposure to HBV and first detection of HBV DNA in serum.

§§ Adjusted to the 2000 U.S. standard population.

¶¶ The trend toward increasing deaths from stimulants and opioids continued into 2019. https://www.cdc.gov/mmwr/volumes/70/wr/mm7006a4.htm?s_cid=mm7006a4_w
